# Navigating the landscape of legal medicine: a 4-year analysis of forensic consultations in an Italian hospital

**DOI:** 10.3389/fmed.2025.1521195

**Published:** 2025-04-30

**Authors:** Paolo Visci, Gianmarco Sirago, Annachiara Vinci, Francesco Calò, Francesco De Micco, Marcello Benevento, Biagio Solarino, Alessandro Dell’Erba, Davide Ferorelli

**Affiliations:** ^1^Section of Legal Medicine Interdisciplinary, Department of Medicine (DIM), University of Bari “Aldo Moro,” Bari, Italy; ^2^Bioethics and Humanities Research Unit, Campus Bio-Medico University of Rome, Rome, Italy; ^3^Department of Clinical Affairs, Campus Bio-Medico University Hospital Foundation, Rome, Italy

**Keywords:** legal medicine, forensic consultations, informed consent, personal injury, sexual violence, patient autonomy, clinical risk management, artificial intelligence

## Abstract

**Introduction:**

This study explores a comprehensive 4-year retrospective analysis of 511 forensic consultations conducted at “Policlinico” hospital in Bari, Italy. It highlights the expanding role of legal medicine within healthcare settings, an area that has traditionally been limited to expert testimony and forensic pathology. Over time, legal medicine in Italy has evolved to address a variety of clinical areas, including informed consent, disability assessment, personal injuries, and sexual violence. This research aims to examine these diverse applications and their impact on patient care.

**Methods:**

Data were systematically categorized and analyzed using a multivariate multinomial regression model. The study focused on key variables, such as patient demographics and timing of shifts, to identify significant determinants that influence the types of forensic consultations conducted. The dataset consisted of 511 consultations, covering a range of clinical and legal issues.

**Results:**

The analysis revealed that informed consent issues were the most prominent, with 58.7% of consultations addressing concerns related to patient autonomy and the capacity for consent, particularly in neuropsychiatric conditions. Personal injury consultations accounted for 24.3% of the total, and sexual assault cases made up 10%. These results underscore the intersection of medical practice and forensic evaluation, highlighting societal issues such as interpersonal violence and the importance of legal medicine in healthcare.

**Discussion:**

The findings highlight significant gaps in the literature regarding the broader applications of legal medicine, especially in terms of integrating advanced methodologies like artificial intelligence. Such technologies could enhance patient profiling and predictive care, ultimately improving patient safety, risk management, and the protection of patient rights. The study advocates for structured forensic consultation services to be incorporated into clinical practice, emphasizing the role of legal medicine in improving patient-centered care and promoting justice. These insights are crucial for healthcare professionals, administrators, and policymakers aiming to optimize healthcare systems.

## 1 Introduction

Legal medicine and forensic science represent the primary interface between medicine and law within healthcare systems. Key components of legal medicine include public health, forensic science, criminology, disability assessment, privacy protection, and ethics. This medical field is interpreted and applied differently across countries worldwide. When the general public is asked, “What is legal medicine?,” responses typically focus narrowly on expert witness roles in court settings (1) and forensic pathology. However, these elements form only a small part of a much broader field that encompasses numerous facets of medical practice, extending its reach into all branches of medicine. This broad applicability is driven by healthcare professionals’ heightened concerns over rising complaints and medico-legal malpractice claims (2).

In recent years, new, particularly pressing areas within legal medicine have emerged. These include defensive medicine—a practice aimed at preventing patient dissatisfaction and avoiding malpractice claims (3)—and clinical risk management, which seeks to enhance the quality and safety of healthcare services (4). In this global context, some countries adopt a holistic approach to forensic and legal medicine, integrating not only forensic pathology and court participation but also criminology, psychopathology, social medicine, and deontology. This comprehensive perspective has led to the establishment of forensic-and-legal-medicine units within hospitals, where these disciplines work collaboratively to meet the requirements of justice administration (5).

Despite the proliferation of forensic and legal medicine units and the myriad medico-legal issues in daily clinical practice, there remains a scarcity of evidence in the literature regarding the scope and nature of tasks undertaken. These gaps represent both a current issue and a future challenge, as these activities address highly sensitive issues, including personal injury, sexual violence, consent and refusal of proposed treatments, and additional clinical complexities. Moreover, structured retrospective data can enable the profiling of patient risk and, prospectively, facilitate a more refined and detailed understanding not only of specific risk profiles but also, with the aid of artificial intelligence, of potential treatment outcomes and associated complications. This article aims to present insights and outcomes from 4 years of forensic consultations within a clinical setting at a major Italian hospital.

## 2 Materials and methods

The study entailed systematically collecting and analyzing forensic consultations conducted over a 4-year period, from 1 January 2019 to 31 December 2022, at the Policlinico Hospital in Bari, which has over 1,500 beds and an Emergency Department. A total of 511 consecutive forensic consultations were gathered, with all data entered into a dedicated database. Consultations involve opinions provided by specialists in a specific medical field to colleagues in other departments regarding the management, treatment, and discharge of admitted patients (6). Following the consultation, the specialist completes a standardized form, routinely included in the patient’s medical record, which documents the consultation outcome. This form typically includes the patient’s details, date and time of consultation, requesting department, diagnostic query, and consultation outcome. These consultations were categorized as summarized in Table 1, and each consultation type was further classified based on various factors, including reported types of sexual assault, discharge protocols, informed consent and capacity for self-determination, personal injury, and other clinical matters.

**TABLE 1:** Data sample.

Variable	Consent	Clinical discharge	Personal injuries	Other clinical issues	Reported sexual assault	Total	*p*-value
Male; *n* (%)	125 (41.95)	7 (28.00)	71 (58.20)	6 (60.00)	4 (7.84)	213 (42.09)	<0.05
Age; mean (SD/range)	71.13(18.98/9–98)	65.68(19.16/15–94)	39,58 (22,07/0–93)	49.89(22.56/16-80)	21.64(14.95/2–68)	57.67(26.48/0-98)	<0.05
Shift; *n* (%)							<0.05
• M	• 158 (52.67)	• 9 (34.62)	• 51 (41.13)	• 6 (60.00)	• 7 (13.72)	• 231 (45.21)	
• A	• 125 (41.67)	• 15 (57.69)	• 45 (36.29)	• 4 (40.00)	• 22(43.14)	• 211 (41.29)	
• N	• 17 (5.66)	• 2 (7.69)	• 28 (22.58)	• 0 (0.00)	• 22(43.14)	• 69 (13.50)	
Requesting unit							<0.05
• Other	• 110 (36.67)	• 15 (57.69)	• 12 (9.68)	• 3 (30.00)	• 10 (19.61)	• 150 (29.35)	
• Plastic surgery	• 1 (0.33)	• 0 (0.00)	• 11 (8.87)	• 0 (0.00)	• 0 (0.00)	• 12 (2.35)	
• Internal medicine	• 38 (12.67)	• 5 (19.23)	• 3 (2.42)	• 1 (10.00)	• 0 (0.00)	• 47 (9.20)	
• Neurology	• 5 (1.67)	• 4 (15.39)	• 6 (4.84)	• 3 (30.00)	• 0 (0.00)	• 18 (3.52)	
• Orthopedics	• 119 (39.66)	• 0 (0.00)	• 2 (1.61)	• 0 (0.00)	• 0 (0.00)	• 121 (23.68)	
• ED	• 26 (8.67)	• 2 (7.69)	• 70 (56.45)	• 3 (30.00)	• 41 (80.39)	• 142 (27.79)	
• Anesthesiology	• 1 (0.33)	• 0 (0.00)	• 20 (16.13)	• 0 (0.00)	• 0 (0.00)	• 21 (4.11)	

This study design is retrospective observational. Data analysis was conducted using Stata MP17 software. Continuous variables were expressed as mean (SD/range), while categorical variables were expressed as proportions. The normality of continuous variables was assessed through skewness and kurtosis tests, although non-normally distributed variables could not be transformed to normality. The Dunn test, with Bonferroni correction for pairwise comparisons, was applied for comparing continuous variables across multiple groups, while the chi-square test was used for comparing categorical variables among multiple groups. Univariate multinomial regression was performed to assess determinants of consultation type, using consent-related consultations as dummy variables and including age, sex, and shift type as determinants. Relative Risk Ratios (RRR) were calculated with 95% confidence intervals (95% CI) provided. A *p*-value of < 0.05 was considered statistically significant for all tests.

## 3 Results

The sample analyzed comprises 511 cases undergoing forensic consultation. Data analysis reveals distribution across five consultation categories: informed consent and capacity for self-determination (300 cases, 58.71%); personal injury (124 cases, 24.27%); reported sexual assault (51 cases, 9.98%); clinical discharge (26 cases, 4.89%); and other clinical issues (10 cases, 2.15%). Among the 511 consultations, 121 were requested by the Orthopedics and Traumatology department (23.68%), and 142 by the Emergency Department (27.79%). Consultations involved male patients in 213 cases (42.09%) and female patients in 298 cases (57.91%), with an average patient age of 57.67 years. This average varied significantly across consultation categories: 71.13 years for informed consent and self-determination, 65.68 years for clinical discharge, 39.58 years for personal injury, 49.89 years for other clinical issues, and 21.64 years for reported sexual assault. Consultations occurred in 231 cases during the morning shift (45.21%), 211 in the afternoon (41.29%), and 69 at night (13.50%). Detailed sample characteristics by consultation type are provided in Table 1.

### 3.1 Informed consent and capacity for self-determination

This was the most frequent consultation type over the 4-year period. Of these, 52.67% were conducted in the morning, and 39.66% were requested by the Orthopedics department. Studied characteristics included the presence of neuropsychiatric conditions, intervention type, consent capacity, the involvement of a support administrator or proxy, and the consultation’s outcome. Cognitive decline accounted for neuropsychiatric conditions in 59.70% of cases. In 32.98% of cases, the intervention involved femoral fracture reduction. Consent capacity was preserved in 24.05% of cases; however, the proposed treatment was carried out without patient consent in 75.44% of cases due to emergency conditions and the necessity of the treatment. Details on informed consent and self-determination issues are summarized in Table 2.

**TABLE 2:** Informed consent data.

Data; *n* (%)	Missing data; *n* (%)	*n*	%
**Neuropsychiatric pathologies**			
• Cognitive decline	32 (10.67)	• 160	• 59.70
• Psychiatric disorders		• 33	• 12.30
• None		• 75	• 28.00
**Type of intervention**			
• Other procedure	18 (6.00)	• 156	• 55.32
• Reduction of a femoral fracture		• 93	• 32.98
• Diagnostic imaging examination		• 18	• 6.38
• Blood transfusion		• 15	• 5.32
Patient’s capacity to express consent	9 (3.00)	70	24.05
Support administrator for healthcare purposes	1 (0.33)	11	3.68
Proxy to third parties	80 (26.67)	6	2.73
**Decision**			
• Treatment carried out without the patient’s consent due to the emergency/urgent conditions	19 (6.33)	• 212	• 75.44
• Treatment with patient’s consent		• 32	• 11.39
• patient’s refusal respected—capable of self-determination		• 22	• 7.83
• Psychiatric evaluation		• 7	• 2.49
• Advance directives previously expressed		• 7	• 2.49
• Other procedure		• 1	• 0.36

### 3.2 Personal injuries

Consultations for personal injuries represented 24.27% of the sample. These occurred during the morning shift in 41.13% of cases, the afternoon in 36.29%, and at night in 22.58%. The Emergency Department requested 56.5% of these consultations. A blunt object was involved in 41.57% of cases, with assault suspected in 74.23% of cases. Injuries were categorized as bruises (34.05%), abrasions (20.54%), and puncture/cut injuries (15.68%). In 90.48% of cases, the aggressor was male, with a family or romantic relationship with the victim in 59.09% and 31.81% of cases, respectively. In 22.58% of cases, injuries were inflicted by a group. Table 3 presents detailed characteristics of personal injury consultations.

**TABLE 3:** Personal injuries data.

Data; *n* (%)	Missing data; *n* (%)	*n*	%
**Mean**			
• Blunt object	35 (28.23)	• 37	• 41.57
• Sharp weapon		• 24	• 26.97
• Firearm		• 14	• 15.73
• Improper weapon		• 6	• 6.74
• Traffic accident		• 4	• 4.49
• Heat-related injury		• 2	• 2.25
• Substance abuse		• 2	• 2.25
**Suspected origin**			
• Assault	27 (21.77)	• 72	• 74.23
• Accidental event		• 16	• 16.49
• Attempted suicide		• 9	• 9.28
**Place where the injuries were made**			
• Home	69 (55.65)	• 25	• 45.45
• Road		• 25	• 45.45
• Public place		• 5	• 9.10
**Type of lesion**			
• Bruises	9 (72.6)	• 63	• 34.05
• Abrasions		• 38	• 20.54
• Puncture and cut injuries		• 29	• 15.68
• Laceration-contusion wounds		• 17	• 9.19
• Bone fractures		• 15	• 8.11
• Firearm		• 13	• 7.03
• Defense-related injuries		• 5	• 2.70
• Thermal agent		• 2	• 1.08
• Absent		• 2	• 1.08
• Poisoning		• 1	• 0.54
Male aggressor	51 (70.83)	19	90.48
**Relationship with aggressor**			
Family member	50 (69.44)	• 13	• 59.09
• Romantic relationship		• 7	• 31.81
• Acquantainance		• 1	• 4.55
• No relationship		• 1	• 4.55
**Number of aggressors**			
• Single	41 (56.94)	• 24	• 77.42
• Group		• 7	• 22.58

### 3.3 Reported sexual assault

Reported sexual assault accounted for 9.98% of consultations, with 68.63% reported as completed assaults. Over 80% of these consultations were requested by the Emergency Department, with a male aggressor involved in 92.16% of cases. A romantic relationship with the victim was present in 13.04% of cases, while the aggressor was an acquaintance in 39.13% of cases. No genital injuries were found in 76.48% of cases; the remaining 23.52% showed genital injuries (5.88% to labia majora, 3.92% perineal, 3.92% urethral, 3.92% hymenal). Other body regions presented no injuries in 48.15% of cases, while bruises appeared in 37.04%. Group assaults accounted for 29.17% of cases, while single aggressors accounted for 21.8%. Table 4 details characteristics of consultations related to reported sexual assault.

**TABLE 4:** Reported sexual assault data.

Data; *n* (%)	Missing data; *n* (%)	*n*	%
**Attempted/consummated**			
• Consummated	0 (0.00)	• 35	• 68.63
• Uncertain		• 12	• 23.53
• Attempted		• 4	• 7.84
Male aggressor	16 (31.37)	35	100
**Relationship with aggressor**			
• Acquantainance	28 (54.90)	• 9	• 39.13
• No relationship		• 6	• 26.09
• Family member		• 5	• 21.74
• Romantic relationship		• 3	• 13.04
**Genital injuries**			
• Absent	2 (3.92)	• 39	• 76.48
• Labia majora		• 3	• 5.88
• Perineal		• 2	• 3.92
• Urethral		• 2	• 3.92
• Hymenal		• 2	• 3.92
• Other		• 3	• 5.88
**Other body region**			
• Absent	1 (1.96)	• 26	• 48.15
• Bruises		• 20	• 37.04
• Lacerations		• 6	• 11.11
• Suction lesions		• 1	• 1.85
• Burns		• 1	• 1.85
Multiple aggressor	25 (49.02)	7	29.17
Use of drugs on the victim	11 (21.57)	10	25

### 3.4 Clinical discharge

Clinical discharge issues constituted 4.9% of consultations. Assessed variables included the reason for hospitalization, presence of prior neuropsychiatric diagnoses, any prior neurological/psychiatric consultations, discharge type, and presence of a support administrator. Among patients, 47.06% had psychiatric disorders, 41.18% had cognitive decline, and 11.76% had intellectual disabilities. Neurological and psychiatric consultations were requested in 64% of cases, with 61.54% of discharges being protected.

### 3.5 Other clinical issues

Consultations for other clinical issues totaled 10 cases, with assessments based on the reason for hospitalization, type of treatment, and outcome. In 80% of cases, the query related to clinical risk management, with 30% hospitalized for gynecological pathologies and another 30% for cognitive decline. Psychiatric therapy was administered in 37.5% of cases, and 33.33% resulted in corporate suicide prevention procedures.

### 3.6 Multivariate multinomial regression

Table 5 summarizes the findings from the multivariate multinomial regression model on the determinants of forensic consultations. Statistically significant results (*p* < 0.05) emerged in the following regressions:

**TABLE 5:** Analysis of determinants of forensic consultation in a multivariate multinomial regression model.

	RRR	95% CI	*p*-value
**Clinical discharge vs. consent**			
Age	0.98	0.96–1.01	0.119
Sex (male vs. female)	0.44	0.17–1.13	0.089
Shift			
• Afternoon vs. morning	1.87	0.78–4.51	0.161
• Night vs. morning	1.73	0.32–9.20	0.521
**Personal injuries vs. consent**			
Age	0.94	0.93–0.95	<0.05
Sex (male vs. female)	1.05	0.62–1.80	0.851
Shift			
• Afternoon vs. morning	1.10	0.63–1.94	0.738
• Night vs. morning	1.94	0.85–4.40	0.114
**Clinical issues vs. consent**			
Age	0.96	0.93–0.98	0.003
Sex (male vs. female)	1.91	0.45–8.05	0.397
Shift			
• Afternoon vs. morning	0.60	0.14–2.46	0.468
• Night vs. morning	0.01	0.01–0.01	0.977
**Reported sexual assault vs. consent**			
Age	0.89	0.87–0.92	<0.05
Sex (male vs. female)	0.06	0.02–0.20	<0.05
Shift			
• Afternoon vs. morning	3.48	1.14–10.67	0.029
• Night *vs.* morning	8.67	2.38–31.54	0.001

• Between “injuries” and the dummy variable “consent” for age: with increasing age, it was significantly less likely for a forensic consultation to be requested for personal injuries or consent issues, suggesting that older patients are more likely to require interventions for which they cannot legally consent, thus necessitating forensic consultation.
• Between “clinical issues” and “consent” for age: as patient age increases, requests for forensic consultations concerning clinical issues are less likely compared to those for informed consent issues.
• Between “sexual violence” and “consent” across all determinants: younger patients are more likely to require consultations for alleged sexual violence than consent-related issues. Additionally, male patients are more likely to be involved in consent-related consultations, while consultations for alleged sexual violence are more likely during the night shift.

## 4 Discussion

The study highlights a significant gap in the literature concerning the scope and nature of tasks performed within Forensic and Legal Medicine Units. Given the sensitive nature of the cases managed—such as personal injuries, sexual violence, and assessments of capacity for self-determination—the lack of supporting evidence poses a substantial challenge. This study addresses this gap by providing a comprehensive retrospective analysis of forensic consultations, exploring the intricate overlap between forensic and legal medicine and clinical practice. This domain intersects diverse fields including public health, criminology, and ethics, and involves collaboration with law enforcement (7).

Clinical challenges increasingly generate forensic considerations (8), leading to new healthcare initiatives focused on preventing harm to patients and mitigating systemic issues that can increase healthcare costs, such as defensive medicine (9). Hospitals regularly face clinically complex cases requiring a delicate balance between patient and healthcare provider rights (10). This has created a growing reliance on consultations from physicians specialized in legal medicine (11). However, data assessing the impact of medico-legal activities within hospital settings remains limited. Our study illuminates the range and complexity of forensic consultations conducted over 4 years in a major Italian hospital, highlighting contemporary healthcare challenges.

The prevalence of consultations concerning informed consent and capacity for self-determination underscores the crucial role of patient autonomy in clinical practice (12). Our findings show a considerable portion of patients with neuropsychiatric conditions, complicating the acquisition of valid consent. Notably, many interventions were conducted without explicit consent due to urgent clinical needs, demonstrating the challenging balance between patient autonomy and timely care.

In this context, effective communication between healthcare providers and patients—or their legal representatives—is paramount. Clear, compassionate, and legally sound discussions about medical decisions, risks, and alternatives are fundamental in preserving patient rights while ensuring that necessary medical interventions are undertaken. Informed consent is not merely a procedural requirement but a cornerstone of ethical medical practice, reinforcing trust in the healthcare system and reducing medico-legal disputes (13).

Personal injury consultations formed another prominent category, reflecting the societal prevalence of violence-related incidents (14). The analysis characterizes various injury patterns, weapon types, and perpetrator demographics, with a notable portion of injuries resulting from assaults by family members or acquaintances, highlighting the complex dynamics of interpersonal violence in social and familial contexts.

Consultations for reported sexual assault present unique forensic challenges, requiring sensitive evaluation of physical and psychological trauma (15). The study reveals the diversity of circumstances in sexual violence cases, including victim-perpetrator relationships, genital injury presence, and cases involving multiple aggressors. The high incidence of cases involving known perpetrators underscores the importance of addressing interpersonal violence beyond traditional stranger assault paradigms. The regression analysis adds a valuable perspective, showing statistically significant associations between consultation types and determinants, with age, gender, and shift timing notably influencing the likelihood of consultations for personal injuries, clinical issues, and sexual violence compared to consent-related consultations. Age-related trends in consultations for personal injuries further illustrate the influence of age on the likelihood of seeking forensic advice.

These findings emphasize the indispensable role of legal medicine in navigating complex clinical scenarios and protecting patient rights and welfare (16). The establishment of structured forensic consultation services within healthcare settings supports the integration of legal and medical expertise, allowing comprehensive assessment and management of medico-legal issues. Furthermore, retrospective analysis of consultation data provides insights for risk profiling, treatment planning, and quality improvement initiatives in clinical practice. Ongoing research and interdisciplinary collaboration are vital for addressing the evolving challenges and complexities within legal medicine (17).

Despite the valuable insights provided by this study, several limitations should be acknowledged:

• Retrospective design: the study relies on previously collected data, which may be incomplete or inconsistent.
• Single-center study: The findings are based on data from a single hospital (Policlinico Hospital in Bari), limiting generalizability to other institutions, regions, or healthcare systems with different medico-legal structures.
• Lack of longitudinal follow-up: The study does not track patient outcomes or the long-term impact of forensic consultations on legal proceedings or healthcare quality.

One key outcome of our study was the detailed analysis of forensic consultations in the orthopedics department, particularly concerning patients with femoral fractures who lacked decision-making capacity. This led to the development of a hospital-wide diagnostic-therapeutic care pathway (PDTA), collaboratively designed by 11 different departments, to ensure integrated management of patients over the age of 65 with femoral fractures. Within this framework, specific provisions were made to align with Italian Law, ensuring that informed consent is obtained before surgical intervention and outlining clear guidelines for cases where patients are unable to provide valid consent. Following the implementation of this PDTA, we observed a marked reduction in requests for medico-legal consultations regarding the management of these patients, demonstrating the tangible impact of forensic clinical medicine on hospital workflows and patient care.

Future studies that utilize advanced methodologies, including artificial intelligence, hold promise for enhancing predictive capabilities and optimizing patient outcomes in the medico-legal domain (18). By embracing an interdisciplinary and innovative approach, healthcare systems can navigate the complex intersection of medicine and law, ultimately promoting justice, equity, and patient-centered care. Understanding the determinants and patterns of forensic consultations has practical implications, guiding healthcare professionals, administrators, and policymakers in developing targeted interventions, enhancing patient safety, and reducing medico-legal risks.

## Conclusion

In conclusion, this article provides an in-depth overview of legal medicine practices, filling an essential gap in the literature. The study not only presents a retrospective analysis of 4 years of forensic consultations but also offers a future-oriented perspective on integrating artificial intelligence in predicting treatment outcomes. The findings underscore the need for a global, unified approach to legal medicine that recognizes its expanding and critical role in contemporary healthcare systems (19).

## Data Availability

The original contributions presented in this study are included in this article/supplementary material, further inquiries can be directed to the corresponding authors.
